# Non-deacetylated poly-*N*-acetylglucosamine-hyperproducing *Staphylococcus aureus* undergoes immediate autoaggregation upon vortexing

**DOI:** 10.3389/fmicb.2022.1101545

**Published:** 2023-01-09

**Authors:** Shoko Kutsuno, Ikue Hayashi, Liansheng Yu, Sakuo Yamada, Junzo Hisatsune, Motoyuki Sugai

**Affiliations:** ^1^Antimicrobial Resistance Research Center, National Institute of Infectious Diseases, Tokyo, Japan; ^2^Department of Antimicrobial Resistance, Hiroshima University Graduate School of Biomedical & Health Sciences, Hiroshima, Japan; ^3^Research Facility, Hiroshima University Faculty of Dentistry, Hiroshima, Japan; ^4^Department of Medical Technology, Faculty of Health Sciences & Technology, Kawasaki University of Medical Welfare, Okayama, Japan

**Keywords:** biofilm, *Staphylococcus aureus*, *icaB*, aggregation, poly-*N*-acetylglucosamine, PNAG

## Abstract

Biofilms are microbial communities of cells embedded in a matrix of extracellular polymeric substances generated and adhering to each other or to a surface. Cell aggregates formed in the absence of a surface and floating pellicles that form biofilms at the air-liquid interface are also considered to be a type of biofilm. *Staphylococcus aureus* is a well-known cause of biofilm infections and high-molecular-weight polysaccharides, poly-*N*-acetylglucosamine (PNAG) is a main constituent of the biofilm. An *icaADBC* operon comprises major machinery to synthesize and extracellularly secrete PNAG. Extracellular PNAG is partially deacetylated by IcaB deacetylase, and the positively charged PNAG hence interacts with negatively charged cell surface to form the major component of biofilm. We previously reported a new regulator of biofilm (Rob) and demonstrated that Rob binds to a unique 5-bp motif, TATTT, present in intergenic region between *icaADBC* operon and its repressor gene *icaR* in Yu et al. The deletion of the 5-bp motif induces excessive adherent biofilm formation. The real function of the 5-bp motif is still unknown. In an attempt to isolate the 5-bp motif deletion mutant, we isolated several non-adherent mutants. They grew normally in turbid broth shaking culture but immediately auto-aggregated upon weak vortexing and sedimented as a lump resulting in a clear supernatant. Whole genome sequencing of the mutants identified they all carried mutations in *icaB* in addition to deletion of the 5-bp motif. Purification and molecular characterization of auto-aggregating factor in the culture supernatant of the mutant identified that the factor was a massively produced non-deacetylated PNAG. Therefore, we created a double deficient strain of biofilm inhibitory factors (5-bp motif, *icaR*, *rob*) and *icaB* to confirm the aggregation phenomenon. This peculiar phenomenon was only observed in Δ5bpΔ*icaB* double mutant but not in Δ*icaR* Δ*icaB or* Δ*rob*Δ*icaB* mutant. This study explains large amount of extracellularly produced non-deacetylated PNAG by Δ5bpΔ*icaB* double mutation induced rapid auto-aggregation of *S. aureus* cells by vortexing. This phenomenon indicated that *Staphylococcus aureus* may form biofilms that do not adhere to solid surfaces and we propose this as a new mechanism of non-adherent biofilm formation of *S. aureus*.

## Introduction

*Staphylococcus aureus* is a facultative anaerobic gram-positive coccus indigenous to human skin, pharynx, fecal, and nasal mucosa (Lowy, 1998; Claassen-Weitz et al., 2016; Mehraj et al., 2016; Byrd et al., 2018). *S. aureus*, the most common cause of nosocomial infections, accounts for a high percentage of isolates from hospitalized patients. *S. aureus* causes a variety of infections, including chronic biofilm infections, such as catheter bloodstream infections, osteomyelitis, and endocarditis. Such biofilm infections are caused by the persistent attachment of *S. aureus* to host tissues, such as bone and heart valves, and to implanted materials, such as catheters and prostheses (Parsek and Singh, 2003; Otto, 2008; Kiedrowski and Horswill, 2011; Barrett and Atkins, 2014; Di Domenico et al., 2022). Biofilm-forming bacteria are more resistant to host defense mechanisms and drugs than non-biofilm-forming bacteria, and the removal of biofilms from indwelling catheters and artificial organs using drugs and immune cells tends to be difficult, resulting in a strong tendency for infections to become refractory (Chatterjee et al., 2014). Biofilms are defined as adherent microbial communities in which cells adhere to surfaces and other cells and are encased in a protective extracellular polymeric matrix (Costerton et al., 1978; Vaccari et al., 2017; Trunk et al., 2018). This mode of growth exhibits altered physiology with respect to gene expression and protein production (Archer et al., 2011; Schilcher and Horswill, 2020). The extracellular matrix is the basis for the attachment of bacteria to the surface of objects and is also involved in the binding of bacteria to each other (Flemming and Wingender, 2010). Its matrix is composed of various components such as nucleic acids, polysaccharides, proteins, and lipids, and the ratio of these components varies with environmental factors among strains as well as species (Mayer et al., 1999; Li et al., 2016). Therefore, the amounts of biofilms as well as their physicochemical and biochemical properties vary among different strains of the same bacterial species.

In general, the major components of staphylococcal biofilms are recognized as high-molecular-weight polysaccharides (poly-*N*-acetylglucosamine, PNAG or polysaccharide intercellular adhesion, PIA in *S. epidermidis*; Mack et al., 1996; Cue et al., 2012), surface protein (Foster et al., 2014), and eDNA (Montanaro et al., 2011; Ibáñez de Aldecoa et al., 2017). These facilitate the attachment of bacterial cells to the surface of objects, followed by bacterial colonization (Lister and Horswill, 2014). The process of PNAG/PIA formation on the cell surface has been well studied using the *S. epidermidis* model (Mack et al., 1996; Vuong et al., 2004; Le et al., 2018). As an extracellular polysaccharide, partially deacetylated PNAG has been obtained from a variety of bacterial sources such as *Escherichia coli* (Wang et al., 2004), *Klebsiella pneumoniae* (Chen et al., 2014), and *Acinetobacter baumannii* (Choi et al., 2009).

PNAG of *Staphylococcus* sp. is achieved *via* a combination of four gene products, *icaA*, *icaD*, *icaB*, and *icaC,* which are tandemly encoded on the *ica* operon of the chromosome (Heilmann et al., 1996a; Cramton et al., 1999). During *ica* operon-induced synthesis of PNAG, *N*-acetylglucosamine undergoes polymerization *via* the combined function of IcaA and IcaD, and then, the resulting *N*-acetylglucosamine polymers are exported *via* the IcaC transporter (Gerke et al., 1998). Exported *N*-acetylglucosamine polymers are partially deacetylated *in situ* by IcaB, and the products accumulate on the cell surface as PNAG (Vuong et al., 2004; Pokrovskaya et al., 2013). In the *S. epidermidis* model, extracellular IcaB partially deacetylated PNAG; subsequently, the positively charged deacetylated forms interacted with the negatively charged cell surface *via* electrostatic interactions and accumulated on the cell surface (Pokrovskaya et al., 2013). Several transcription factors are involved in the regulation of PNAG (Conlon et al., 2002; Jefferson et al., 2004; You et al., 2014; Yu et al., 2017). IcaR, located just upstream of the *icaADBC* operon, is a well-studied negative regulator that suppresses the *icaADBC* operon (Conlon et al., 2002; Jefferson et al., 2004). The product of *rob* (Yu et al., 2017)*,* which is a newly discovered negative regulator (also known as GbaA (You et al., 2014)), binds to a 5-bp motif (TATTT) in the *icaR*-*icaA* intergenic region and suppresses the expression of *icaADBC*. The 5-base motif and *rob* are not present in *S. epidermidis*; thus, *rob* is a unique negative regulator of *icaADBC* in *S. aureus*. Jefferson et al. (2003) demonstrated that a clinically identified *S. aureus* strain, which produced a large amount of biofilm, lacked the 5-base motif, suggesting its importance in the regulation of biofilm production (Jefferson et al., 2003). It remains unclear whether the product of *rob* is the only regulator capable of recognizing the 5-base motif.

In order to further analyze the physiological function of the 5-base motif, a 5-base motif deletion mutant of *S. aureus* FK300, poor biofilm forming strain, was generated. Although most mutants showed normal colony morphology, a few revealed peculiar phenotypes, as indicated by colonies showing shiny flat morphology and fusion with adjacent colonies.

These colonies grew normally in turbid broth shaking culture but immediately autoaggregated upon weak vortexing and sedimented as a lump resulting in a clear supernatant. These mutants completely lacked the ability to form biofilms on the surface.

In this study, we investigated the molecular mechanism underlying this autoaggregation just after the vortexing of mutants with a 5-base deletion.

## Materials and methods

### Bacterial strains and growth media

In this study, the shaking culture was performed in a water bath using a test tube of φ15 mm × 150 mm with shaking at 140 rpm at an angle of 45°. The bacterial strains and plasmids used in this study are listed in Supplementary Table 1. Standard strain *S. aureus* FK300, a *rsbU*-repaired (Giachino et al., 2001) derivative of NCTC8325-4 (Novick, 1967; Herbert et al., 2010), was used in a functional study of the role of *icaB*. DNA restriction system-deficient *S. aureus* RN4220 (Kreiswirth et al., 1983) was used as the initial recipient for manipulation of recombinant plasmids. *S. aureus* was routinely grown in brain heart infusion (Becton, Dickinson and Company, MD, United States) broth, tryptic soy broth (TSB; Becton, Dickinson and Company), or tryptic soy agar plates. Tetracycline (5 μg/ml) or chloramphenicol (5 μg/ml) was added to retain plasmids. The *Escherichia coli* strain DH5α was used for plasmid construction and maintenance. *E. coli* was grown in lysogeny broth (LB; 5 g yeast extract, 10 g polypeptone, and 10 g NaCl per liter; pH 7.2) or LB agar. Ampicillin (100 μg/ml) or tetracycline (12.5 μg/ml) was added to the medium when the plasmid was to be retained.

### Plasmid and strain construction

Routine DNA manipulation was performed as previously described (Sambrook et al., 1989). FK300 mutants were constructed *via* allelic replacement using pKFT (Kato and Sugai, 2011). Polymerase chain reaction (PCR) was performed using TaKaRa LA Taq (TaKaRa, Shiga, Japan). The thermal cycling conditions were as follows: 94°C for 2 min, followed by 30 cycles of 94°C for 15 s, 50°C, for 30 s, and 68°C for 2 min, finishing with a final extension step at 72°C for 1 min. The oligonucleotides used in this study are listed in Supplementary Table 2. Fragments were cloned into the plasmid pGEM-T Easy (Promega, Madison, WI, United States) using TA cloning and transformed into *E. coli* DH5α. A fragment excised from the pKS101 plasmid using a restriction enzyme was cloned into the pKFT plasmid and transformed into *E. coli* DH5α. Recombinant plasmids were introduced into *S. aureus* RN4220 *via* electroporation (Kraemer and Iandolo, 1990; Löfblom et al., 2007; Hisatsune et al., 2016). The modified plasmids were then electroporated into *S. aureus* FK300 cells for allelic replacement. Marker-less deletion mutants in tetracycline-sensitive colonies were screened using PCR. Fragments were verified using DNA sequencing with a BigDye Terminator v 3.1 Cycle Sequencing Kit (Applied Biosystems, Waltham, MA, United States). In the complementation experiments, genes were amplified using PCR with the corresponding primer pairs and then cloned into the SmaI site of pKAT (Kato, 2004). The plasmids, pKS103 and pKS104, carrying *the icaR* and *icaB* coding regions of FK300, respectively, were constructed and transformed into the *S. aureus* strains listed in Supplementary Table 1 using electroporation. Inserts in all plasmid constructs were verified using PCR and DNA sequencing.

### Biofilm assay

A biofilm assay using polystyrene plates was performed as described previously (Heilmann et al., 1996b) with a few modifications. Briefly, overnight cultures were diluted 1:100 in TSB. Ten microliters of this diluted solution was transferred in triplicate into flat-bottom, 96-well polystyrene plates (TrueLine; Nippon Genetics Co., Ltd., Japan) containing 100 μl of TSB or TSB plus 1% glucose. Following incubation at 37°C for 20 h, the wells were gently washed thrice with 320 μl of sterile phosphate-buffered saline (PBS; 137 mM NaCl, 2.7 mM KCl, 10 mM Na_2_HPO_4_·12H_2_O, and 1.8 mM KH_2_PO_4_; pH 7.4), and the biofilm was stained with 1% crystal violet for 15 min. Unbound crystal violet was then removed by washing the plate in a container by immersing and agitating it gently 10 times in tap water and then drying it. Biofilm-bound crystal violet was solubilized in 150 μl of 33% glacial acetic acid at 25°C for 15 min. The extracts were diluted 10-fold, and then, absorbance at 590 nm was measured using an Immuno-Mini NJ-2300 spectrophotometer (Nalgene Nunc International K. K., Tokyo, Japan). Each assay was performed in triplicate and repeated three times.

### Autoaggregation assay

Bacteria from the plate were inoculated into 3 ml of TSB in a test tube (φ15 × 150 mm), placed in a water bath, and shaken at 140 rpm at an angle of 45° for 6 h. The culture was vortexed (Vortex-Genie 2; Scientific Industries, Inc.) for 10 s. Turbidity of the culture was visually monitored using video photography. For swapping experiments, the culture supernatant of interest was passed through a 0.2-μm filter, incubated with the cells of interest, and washed several times with 10 mM PBS; thereafter, the autoaggregation assay was carried out as described above.

### RNA isolation and quantitative real-time reverse transcription-PCR (qRT-PCR)

Real-time PCR of *ica* operon in *S. aureus* has been previously described (Yu et al., 2017). Overnight *S. aureus* cultures were diluted in TSB containing 1% glucose to an initial optical density (OD) of 0.02 at 660 nm and harvested after 6 h of incubation with shaking at 37°C. Total RNA was isolated using a FastRNA Pro Blue kit (MP Biomedicals, Santa Ana, CA, United States) according to the manufacturer’s instructions. DNA was extracted by treatment with RQ1 RNase-free DNase (Promega) at 37°C for 30 min. After DNase inactivation, PCR was performed to verify the absence of contaminating DNA. RNA was then reverse-transcribed using a Transcriptor First-Strand cDNA Synthesis Kit (Roche, Mannheim, Germany). The resulting cDNA was diluted 10-fold with Tris-EDTA buffer (10 mM Tris–HCl and 1 mM EDTA; pH 8.0) and used as a template in the real-time PCR. qRT-PCR was performed using the SsoAdvanced Universal SYBR Green SuperMix (Bio-Rad, Hercules, CA, United States) and a CFX96 real-time PCR detection system (Bio-Rad). The thermal cycling conditions were as follows: 95°C for 1 min, followed by 40 cycles of 95°C for 15 s, 60°C (*icaA*), or 62°C (*gyrB*) for 15 s, and 72°C for 30 s. All PCR runs were performed in triplicate, and data were analyzed using the CFX Manager software (version 3.0; Bio-Rad) according to the manufacturer’s instructions. The housekeeping gene, gyrase subunit B (*gyrB*), was used as a reference gene to normalize the expression level of the target gene in each reaction. The primers used for real-time PCR are listed in Supplementary Table 2. All samples were inspected 3 times and the data were analyzed using the 2^-ΔΔCt^ method (Livak and Schmittgen, 2001).

### Electron microscopy

Electron microscopic observations were performed using TEM, as previously reported (Yamada et al., 1996). Bacterial cells were harvested, washed twice with 0.1 M PBS, and collected using centrifugation (2,300 × *g*, 15 min). For TEM, cells were fixed with 2.5% glutaraldehyde and 1% OsO_4_. Samples were dehydrated using an ethanol series and embedded in New Spurr (Agar Scientific Ltd., United Kingdom). Ultrathin sections were cut with an ultramicrotome (ULTRACUTS, Leica, Tokyo, Japan) and examined with a JEOL JEM-2000 EXII electron microscope (JEOL Ltd., Tokyo, Japan) at 80 or 100 kV.

### Element analysis

Elemental analysis of autoaggregates from the culture supernatant of FK300Δ5bpΔBm was performed using a scanning electron microscope connected to an energy dispersive X-ray spectroscope (Miniscope TM-3030, Hitachi High-Technologies Corporation, Tokyo, Japan).

### Gel permeation HPLC

The culture supernatant was concentrated *via* centrifugation using an Amicon Ultra 15 ml filter (3 kDa cut off, Merck Millipore Corporation, Darmstadt, Germany) for 40 min at 4,000 × *g*, and the concentrate was treated with trichloroacetic acid (final conc. 5%) to remove proteins by centrifugation for 15 min at 6,000 × *g*. Subsequently, the supernatant was subjected to gel permeation chromatography using Shim-pack Diol-300 (500 × 7.9 mm) with water as the mobile phase at a flow rate 0.5 ml/min. The effluent was fractionated every 2 ml from fraction 1–21, 11 min after sample injection. Samples necessary for hydrolysis were treated at 100°C for 2 h in the presence of 2 N HCl and rehydrated with distilled water after evaporation for the colorimetric assay.

### Colorimetric determination of amino sugars

Fractions obtained by HPLC were analyzed for amino sugars using the Morgan-Elson assay (Enghofer and Kress, 1979). Samples (100 μl) were incubated with 20 μl acetone containing 1.5% acetic anhydride and 100 μl boric acid buffer (pH 9.0) at 95°C for 8 min and kept on ice. Then, the samples were mixed with 750 μl of p-(dimethylamino) benzaldehyde containing 12.5% HCl and 50 μl of 2-ethoxyethanol, and left to stand at room temperature for 15 min. OD at 545 nm was measured using a microplate reader (Varioskan LUX multimode reader, Thermo Fisher Scientific, Waltham, MA, United States).

### PNAG dot blot

PNAG dot blotting was performed on each fraction obtained by fractionating the supernatant using gel filtration chromatography (Cramton et al., 1999). Alternatively, Bacteria from the plate were inoculated and incubated in 3 ml TSB medium at 37°C for 6 h with shaking. The incubated bacteria were centrifuged (8,000 g × 2 min), and the supernatant and bacteria were separated. To 1 ml of supernatant passed through a 0.2 μm sterile filter, 50 μl of Proteinase K (50 μl/ml; TaKaRa) was added and allowed to react at 55°C for 30 min, followed by incubation at 85°C for 30 min to inactivate the protease. The bacterial cells were washed once with PBS, resuspended in 100 μl of 0.5 M EDTA, and boiled at 100°C for 5 min. To 40 μl of the supernatant after centrifugation, 10 μl of Proteinase K (50 μl/ml; TaKaRa) was added and reacted at 55°C for 30 min, followed by incubation at 85°C for 30 min to inactivate the protease. The resulting bacterial surface samples and culture supernatant samples were serially diluted. The bacterial cell surface sample, supernatant sample or HPLC fraction was dropped onto a nitrocellulose membrane (Amersham Protran NC 0.45; General Electric Company, Connecticut, United States) and the membrane was immersed in a TBS-T [50 mM Tris–HCl (pH 8.0), 150 mM NaCl, and 0.05% Tween 20 (Sigma-Aldrich, Inc.)] solution containing 5% skim milk and allowed to react for 1 h to block the membranes. The membrane was then washed thrice with TBS-T for 15 min and incubated with rabbit anti- Poly-β-1,6-*N*-acetyl-D-glucosamine (PNAG) antiserum supplied by Dr. Gerald Pier (Skurnik et al., 2010) diluted 1:4,000 with 0.5% skimmed milk in PBS-T for 2 h at room temperature. Bound antibody was detected with peroxidase-conjugated goat anti-rabbit immunoglobulin G (IgG) antibodies (MP Biomedicals, LLC-Cappel Products, Ohio, United States; 1:6,000) and developed using Pierce enhanced chemiluminescence (ECL) western blotting substrate (Thermo Fisher Scientific, Rockford, IL, United States). Results were detected using a charge-coupled device camera-based imager (GE Healthcare Life Sciences, Piscataway, NJ, United States) or FujiRX-U film (Fujifilm, Tokyo, Japan), and developed using HI-RENDOL (Fujifilm) and HI-RENFIX (Fujifilm).

### Electrospray ionization mass spectrometry (ESI-MS) analysis

MS analysis was performed using an LTQ Orbitrap XL mass spectrometer (Thermo Fisher Scientific) on a direct infusion MS using a nanospray capillary.

Samples in 50% methanol containing 0.1% formic acid were ionized using electrospray ionization in positive ion mode, and the following parameters were set: spray voltage, 1.5 kV; tube lens voltage, 250 V(max); source fragmentation, ON (100 Vmax); and mass acquisition range, m/z 200–4,000. Data were processed using the Xcalibur software package provided by Thermo Fisher Scientific.

### Statistical analysis

Differences between two means were evaluated by the Mann–Whitney U test. Data were analyzed by Kruskal-Wallis test of variance to compare multiple means. Differences with *p*-values less than 0.05 were considered statistically significant. Statistical analysis was performed using R 4. 1.3 was used, with a significance level of 5%.

## Results

### Effects of *icaB* on cell autoaggregation and biofilm elaboration of *Staphylococcus aureus* FK300

**Figure 1:**
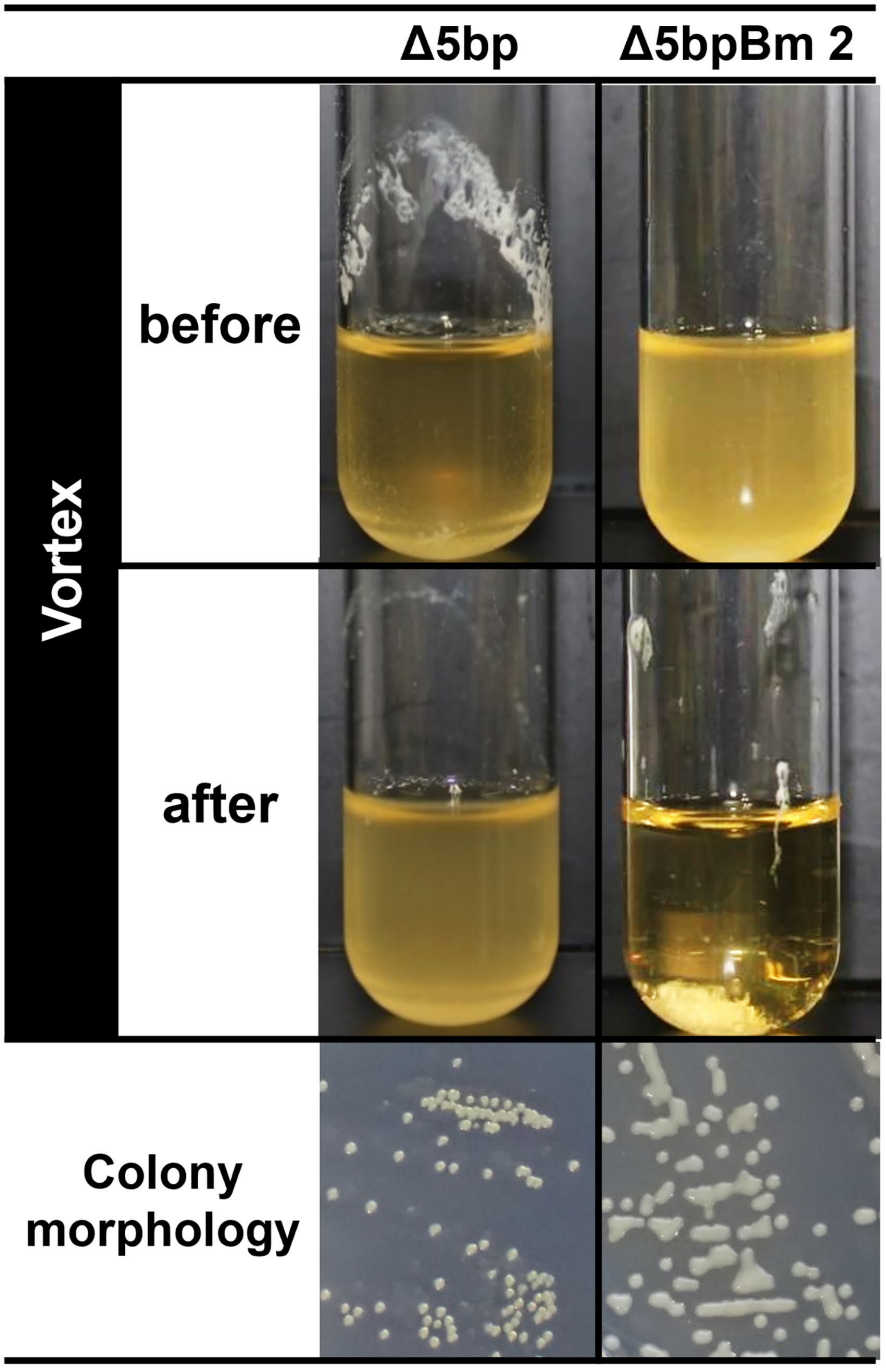
Autoaggregation assay of *S. aureus* FK300 5 bp deletion mutant culture. Autoaggregation assay of *S. aureus* FK300 5-bp deletion mutant culture. During the process of isolating the 5-bp (*icaR*-*icaA* intergenic region) deletion mutant from *S. aureus* FK300, we obtained colonies showing a distinct phenotype and the isolates were designated as Δ5bpBm 1, 2, and 3. These isolates were cultured in TSB for 6 h (before), and vortexed for 10 s (after), following which images of the culture were obtained (Supplementary Video 1). Shown is an image of the shape of the colonies growing on TSA plate. Left, genuine Δ5bp mutant; right, Δ5bpBm 2. Other Δ5bpBm mutants revealed a similar phenotype (Supplementary Figure 1).

The deletion of the 5-bp motif mutant from *S. aureus* wild type (WT) strain FK300 *via* allelic replacement resulted in the isolation of three independent mutants (Δ5bpBm 1, 2, and 3) showing a common but unusual phenotype (Supplementary Figure 1). Most isolates upon 5-bp deletion mutation showed normal colony morphology, but a few revealed unusual phenotypes, as indicated by colonies showing shiny flat morphology, compounded by fusion between adjacent colonies (Figure 1, colony). In broth culture, most possible 5-bp motif-deletion mutants revealed a super-biofilm-producing phenotype, where these mutants exhibited thick pellicles in test tube broth culture, as observed in a previous study (You et al., 2014; Figure 1, left, Δ5bp, before vortex). By contrast, the three isolates in our study did not show any pellicles on the wall of the test tube (Figure 1, right, Δ5bpBm 2, before vortex). Furthermore, weak mixing using a vortex mixer for a few seconds induced instant autoaggregation and subsequent sedimentation of the bacteria, resulting in a clear supernatant (Figure 1, right, Δ5bpBm 2, after vortex), whereas the genuine mutant Δ5bp remained turbid (Figure 1, left, Δ5bp, after vortex; Supplementary Video 1).

**Figure 2:**
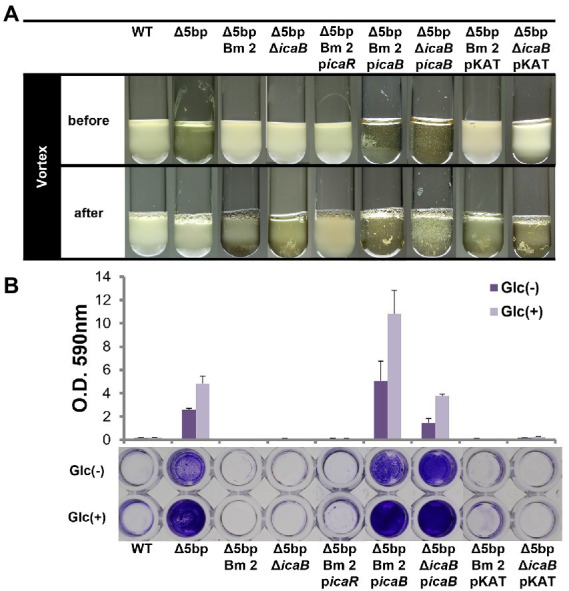
Dysfunction of IcaB and advanced biofilm production cause aggregation. Combination of IcaB dysfunction and 5-bp deletion cause autoaggregation. Autoaggregation assay **(A)** and biofilm assay **(B)** of various *Staphylococcus aureus* FK300 and the associated mutants: FK300Δ5bp; FK300Δ5bpBm 2; FK300Δ5bpΔ*icaB*; and FK300Δ5bpBm 2 complemented with pKAT carrying *icaR* (p*icaR*), *icaB* (p*icaB*), or pKAT, whereas FK300Δ5bpΔ*icaB* complemented with p*icaB* or pKAT. Autoaggregation assay was carried out as shown in Figure 1. Biofilm assay was conducted using a microtiter plate. Bacteria were grown in TSB in the presence (Glc) or absence (Glc-) of 1% glucose. Biofilm stained with crystal violet was solubilized and OD 590 nm was measured using the polystyrene microtiter plate as described in the Methods section. Bars indicate mean values, error bars indicate standard error of the mean (n = 3). WT, wild type strain FK300. pKAT, empty vector control.

Whole-genome sequencing of the three mutants established that their 5-bp motif was defective, indicating that the deletion of 5-bp had been successful. In addition, we identified a point mutation in the coding sequence of *icaB* in each strain (Supplementary Figure 2). For Δ5bpBm 3, a frameshift mutation occurred due to the insertion of T, resulting in the creation of a termination codon 67 bases downstream, whereas Δ5bpBm 2 and Δ5bpBm 1 demonstrated single and double missense mutations, respectively. Thereafter, FK300Δ5bpBm 2 was used as the representative strain in the experiment. When FK300Δ5bpBm 2 mutant was complemented with *icaB*, it formed adherent biofilms after culture and shown pellicle formation *in vitro* (Figure 2A). After voltexing, the formed biofilm was not completely dissolved as that of Δ5bp probably due to overproduction of adherent biofilm and remained detached was suspended as clumps. Biofilm assays using plastic plates substantiated that FK300Δ5bpBm 2 had completely lost the ability to form adherent biofilms, whereas complementation with *icaB* restored strong biofilm production (Figure 2B). These results suggested that *icaB* was involved in the unusual phenomenon displayed by the three FK300Δ5bpBm 2 mutant. Therefore, we generated FK300Δ5bpΔ*icaB* and verified that the mutant showed the same phenotype as FK300Δ5bpBm 2. This phenotype was also successfully complemented by *icaB*. To establish that this phenomenon was dependent on the *ica* operon, we overexpressed *icaR* in FK300Δ5bpΔ*icaB* to regulate the production of PNAG. FK300Δ5bpBm 2 p*icaR* completely lost the autoaggregating phenotype observed in FK300Δ5bpΔ*icaB*, indicating its dependence on the *ica* operon (Figure 2).

**Figure 3:**
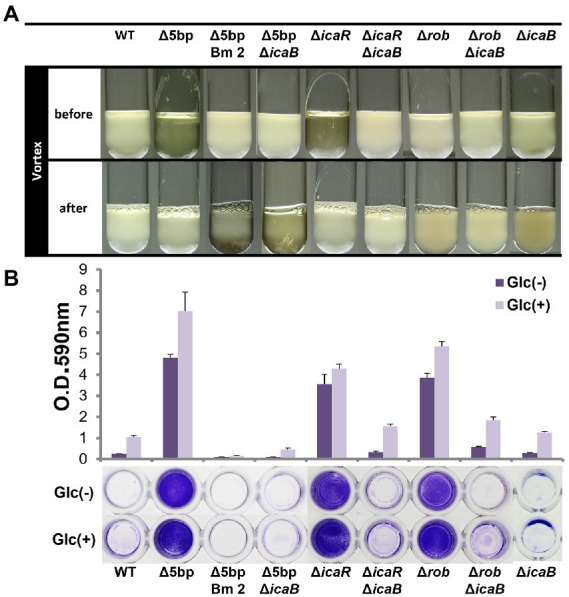
Comparison of each biofilm formation inhibitory factor and *icaB* double deficient strains. Autoaggregation and biofilm formation of Δ5bpΔ*icaB*, Δ*icaR*Δ*icaB,* and Δ*rob*Δ*icaB* double mutants. Autoaggregation assay **(A)** and biofilm formation **(B)** of FK300 and FK300Δ5bp, FK300Δ5bpBm 2, FK300Δ5bpΔ*icaB*, FK300Δ*icaR*, FK300Δ*icaR*ΔicaB, FK300Δ*rob*, FK300Δ*rob*Δ*icaB* and FK300Δ*icaB*. Status before and after mixing for a few seconds on a vortex mixer is shown. Each bacterium was cultured in TSB medium at 37°C for 6 h with shaking. **(B)** Bacteria were grown in TSB in the presence (Glc) or absence (Glc-) of 1% glucose. Biofilm formation was measured using the polystyrene microtiter plate assay as described in the Methods section. Bars indicate mean values, error bars indicate standard error of the mean (*n* = 3). WT; wild type strain FK300.

**Figure 4:**
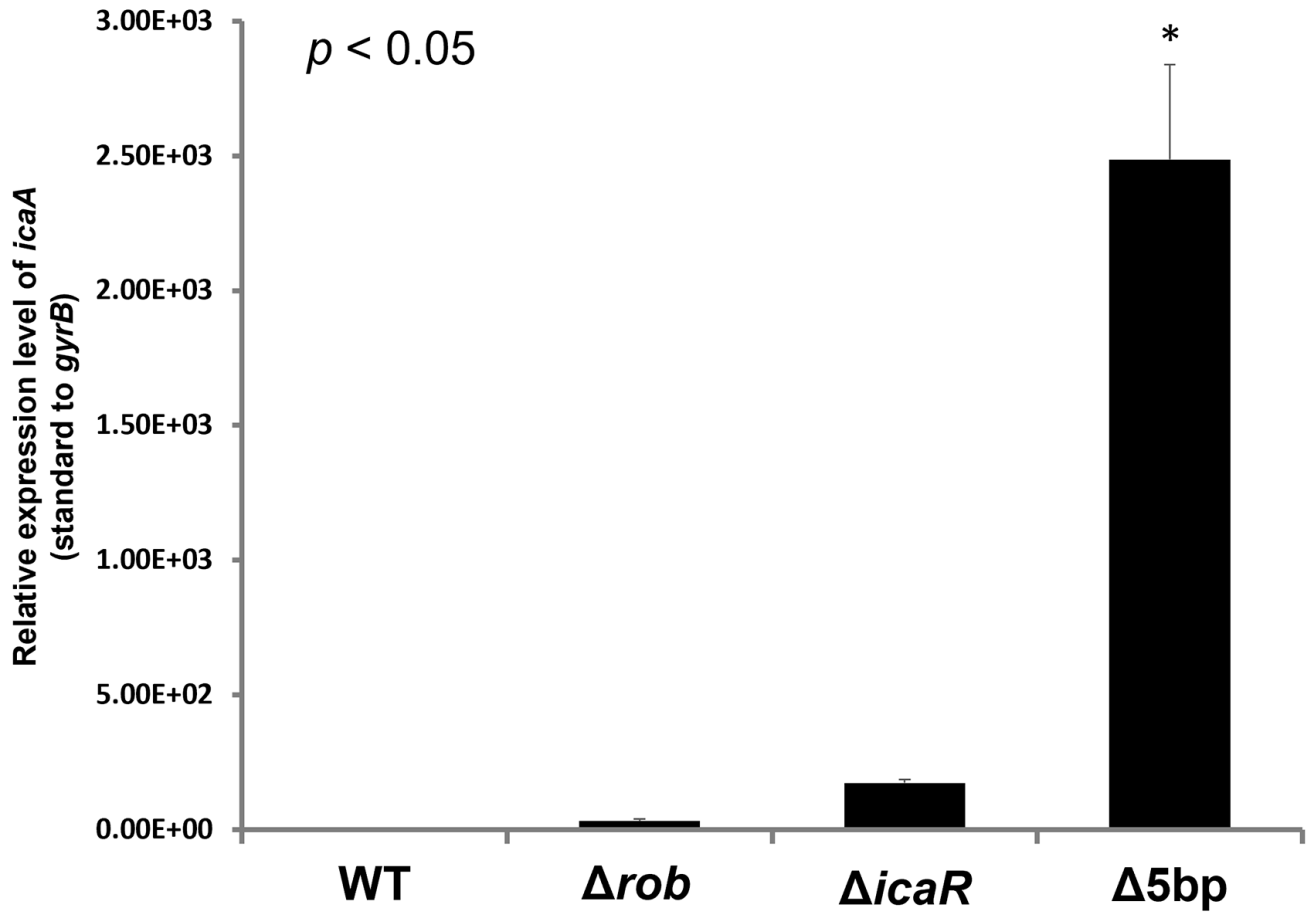
Measurements of *icaA* transcription by qPCR. *Ica* operon expression of hyper-biofilm elaborating mutants. Total RNA preparation, cDNA synthesis, and quantitative PCR were performed as described in the Methods section. Relative expression level of *ica* operon; transcript levels in the Δ5bp, *icaR,* or *rob* deletion mutants as compared to those in WT strain FK300 are shown. The expression of *gyrB* was used for sample normalization. Bars indicate mean values, error bars indicate standard error of the mean (*n* = 3). Kruskal-Wallis test results *p* < 0.05. There were significant differences between all deficient strains and WT. *: *p* < 0.05 (Mann–Whitney U test). WT; wild type strain FK300.

**Figure 5:**
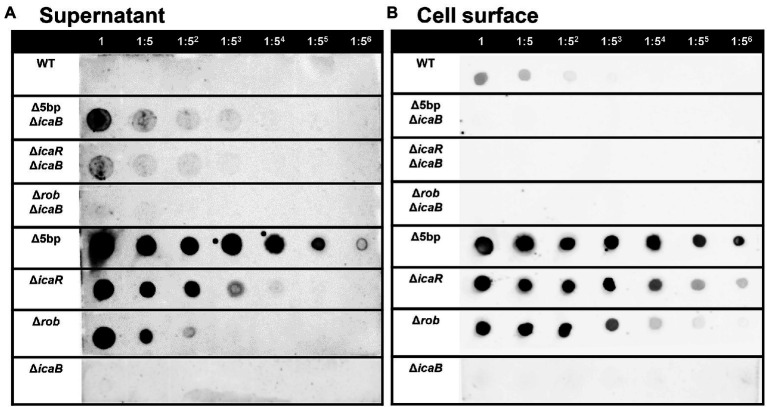
PIA / PNAG production on the culture supernatant and cell surface in each strain. PIA/PNAG production in the culture supernatant and on cell surface of the mutants. PIA/PNAG collected from the culture supernatant and cell surface of FK300 and FK300Δ5bpΔ*icaB*, FK300Δ*icaR*Δ*icaB*, FK300Δ*rob*Δ*icaB*, FK300Δ5bp, FK300Δ*icaR,* FK300Δ*rob* and FK300Δ*icaB* cultured for 6 h were evaluated *via* dot blotting on nitrocellulose using rabbit antibody against PIA. **(A)** supernatant, **(B)** cell surface. WT; wild type strain FK300.

Deletion of the 5-bp motif is known to increase biofilm formation (Yu et al., 2017). However, other regulatory repressor regullators (*rob* and *icaR*) are also involved in biofilm formation, and the deletion of these factors has been shown to increase biofilm formation (Jefferson et al., 2004; Yu et al., 2017). We therefore created *icaB* deletion mutants and double deletion mutants combining the repressor and Δ*icaB* to see if it would develop a self-aggregating phenotype as observed with Δ5bpΔ*icaB*. Although all three deletion mutants lost the adherent biofilm formation ability (Figure 3B), only FK300Δ5bpΔ*icaB* showed an autoaggregating phenotype (Figure 3A). This result was reproduced when the culture was shaken even 250 rpm (Unpublished data). Tiny autoaggregates were observed in the broth of FK300Δ*rob*Δ*icaB* and FK300Δ*icaR*Δ*icaB*, but there were no pellicles and the supernatant remained turbid even after mixing using a vortex mixer. This may likely have been due to the differences between the magnitudes of activation of the *ica* operon in the deletion mutants (Δ*rob*, Δ*icaR*, and Δ5bp), and we, therefore, measured mRNA levels of *ica* operon in the respective mutants. The results showed that expression of *ica* operon by Δ5bp was much stronger than that by Δ*rob* or Δ*icaR* (Figure 4). The *ica* operon expression levels of each defective mutant compared to WT were about 31.46-fold for Δ*rob*, 173.90-fold for Δ*icaR*, and 2486.17-fold for Δ5bp. This is consistent with the report of Yu et al. (2017). Based on these observations, we analyzed fractions of the culture supernatant and cell surface of each strain using anti-PNAG antiserum dot blot in order to investigate the actual production of PNAG. No signal was observed on the cell surfaces of FK300Δ5bpΔ*icaB*, FK300ΔicaRΔ*icaB*, or FK300ΔrobΔ*icaB*. However, in the culture supernatant, a clear strong signal demonstrating a reaction with the anti-PNAG antiserum was seen for FK300Δ5bpΔ*icaB* and a weak one for FK300Δ*icaR*Δ*icaB*, but not for FK300Δ*rob*Δ*icaB* (Figures 5A,B). On the other hand, strong signals were detected in both the culture supernatant and the cell surface in FK300Δ*rob*, FK300Δ*icaR,* and FK300Δ5bp, respectively as expected. For FK300, a very weak signal was observed only at the cell surface, and no signal was detected in the culture supernatant. For FK300Δ*icaB*, no signal was observed in both culture supernatant and cell surface.

### Substances that caused autoaggregation of cells

**Figure 6:**
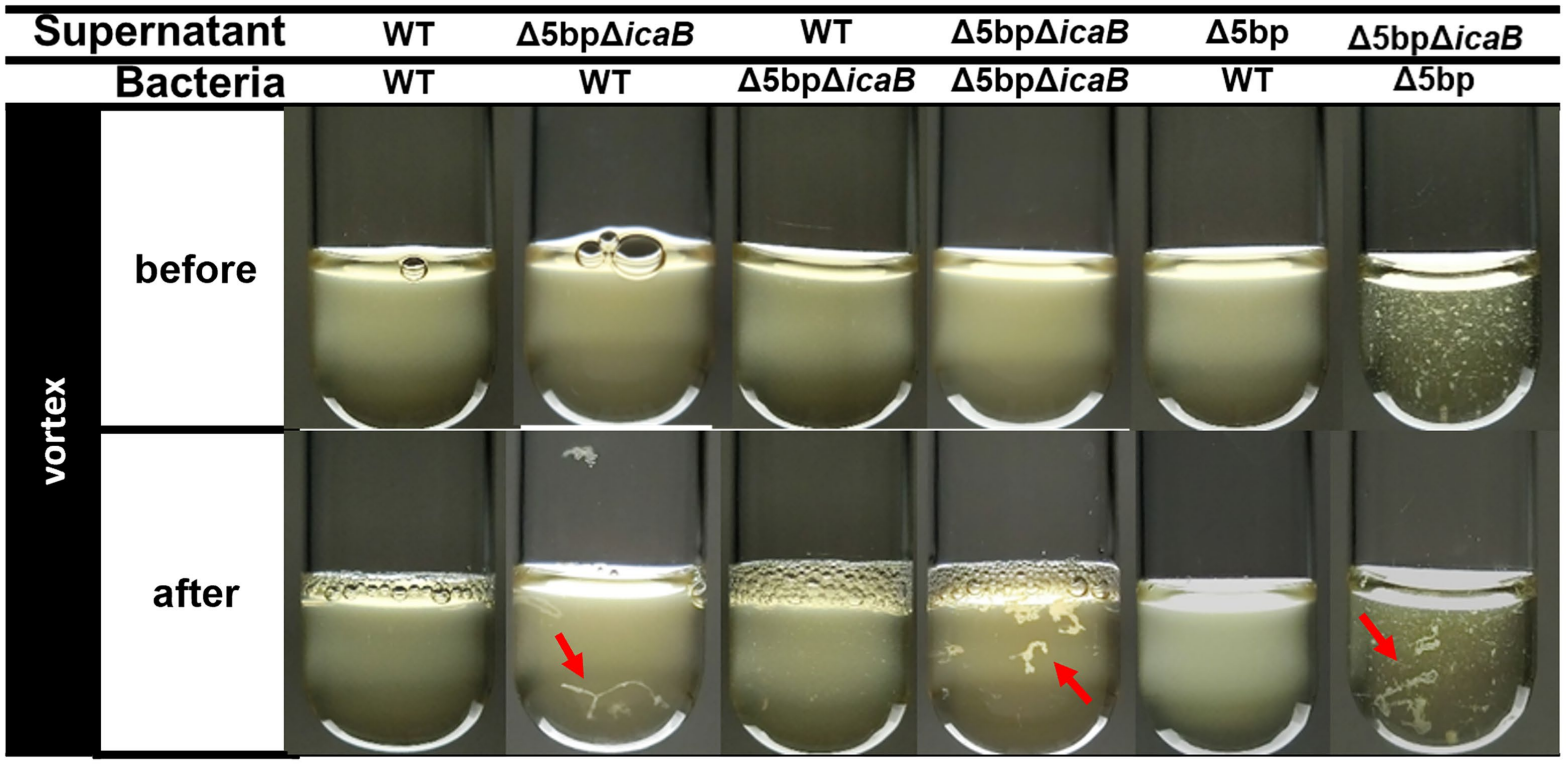
Swapping experiment of culture supernatant and bacterial cells. Swapping of culture supernatant and bacterial cells. Filtered culture supernatant of FK300, FK300Δ5bp, or FK300Δ5bpΔ*icaB* was incubated with bacterial cells of FK300 or FK300Δ5bpΔ*icaB* (before) and vortexed for 10 s (after), and photographic images of the culture were obtained. Aggregates identified after vortexing were indicated by red arrows. WT; wild type strain FK300.

**Figure 7:**
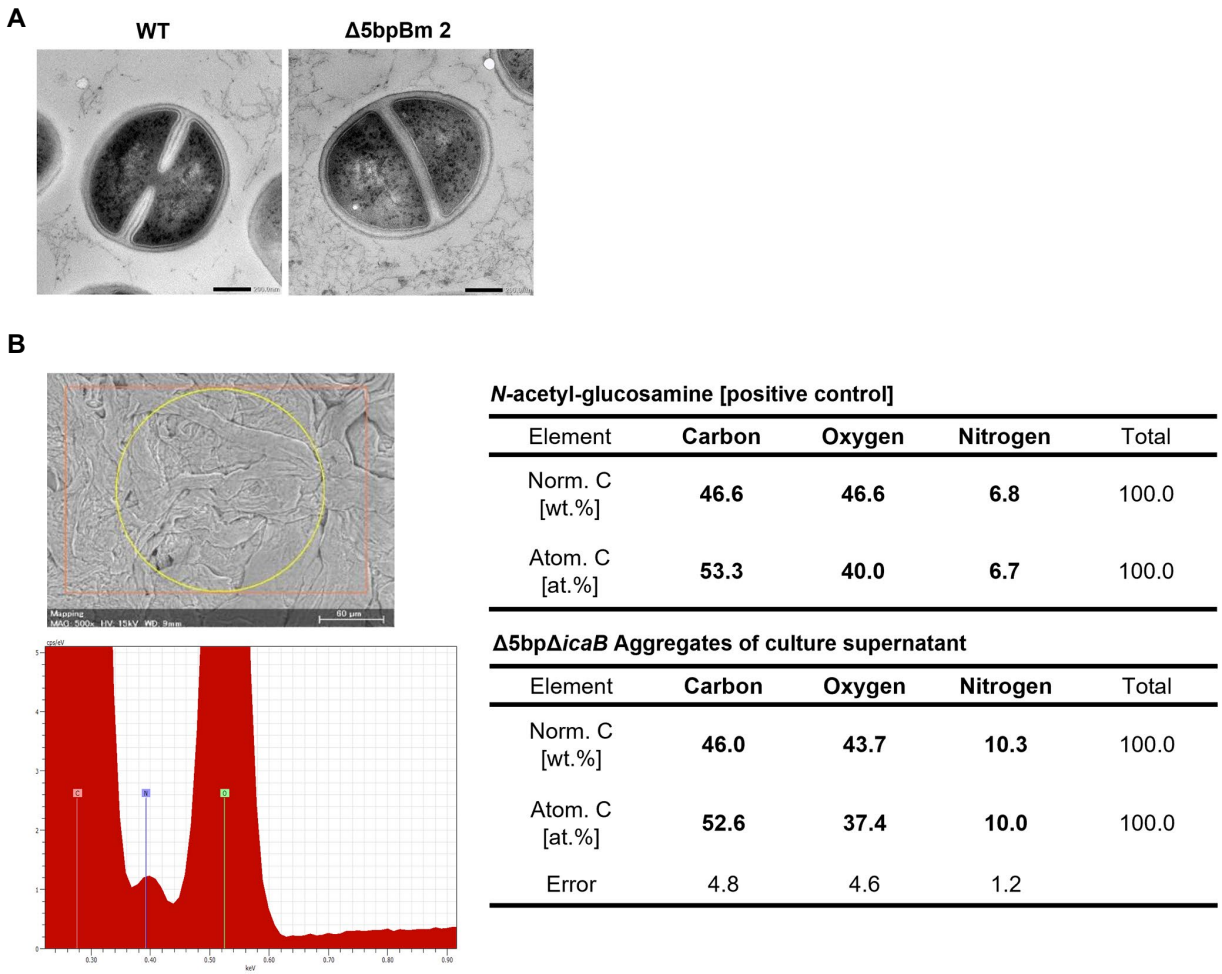
Searching for autoaggregation factors using electron microscopy and elemental analysis. Morphological and elemental analysis of autoaggregation factor(s) using electron microscopy. **(A)** Ultrastructural observation of *Staphylococcus aureus* FK300Δ5bpBm 2 using transmission electron microscope. *S. aureus* FK300 (left) and *S. aureus* FK300Δ5bpBm 2 (right). **(B)** Elemental analysis of the autoaggregate from FK300Δ5bpΔ*icaB* supernatant. Autoaggregates obtained by vortexing the culture supernatant of FK300Δ5bpΔ*icaB* were dried and examined *via* a scanning electron microscope (Mini Scope TM-3030) for element analysis. As a control, the value of *N*-acetylglucosamine is shown. WT; wild type strain FK300. The top left figure is SEM image of a sample irradiated with characteristic X-rays. The bottom left figure is EDS spectrum. The right is a table of elemental ratios of flocculence samples and *N*-acetylglucosamine.

We conducted a swapping experiment to identify the factor(s) involved in autoaggregation on the cell surface or in the culture supernatant of FK300Δ5bpΔ*icaB.* The culture medium of each bacteria was separated into cells and culture supernatant, and a vortex experiment was performed by multiplying the selected supernatant with the selected cells to see which sample was important for aggregation. The strains used in the experiment were FK300, FK300Δ5bp, or FK300Δ5bpΔ*icaB*. The autoaggregating factor(s) was present in the culture supernatant of FK300Δ5bpΔ*icaB*, but not in the culture supernatant of FK300Δ5bp or on the cell surface of FK300Δ5bpΔ*icaB* (Figure 6). The results suggest that the important factor for aggregation is in the culture supernatant of FK300Δ5bpΔ*icaB*. We examined the ultrastructure of the autoaggregates using transmission electron microscopy (TEM). The autoaggregate preparation obtained from sedimented FK300Δ5bpBm 2 was compared with a cell pellet of WT FK300. There was no significant difference between the ultra-structures of the cell wall peptidoglycan layers of the strains (Figure 7A). However, string-like substances surrounding FK300Δ5bpBm 2 were observed, implying the presence of string-like substances in the culture supernatant of FK300Δ5bpBm 2. We further analyzed the elements of the autoaggregates obtained from the culture supernatant of FK300Δ5bpΔ*icaB* using a TM-3030 Hitachi microscope connected to energy dispersive X-ray spectroscope. Dried autoaggregate was spotted using a miniscope, and the spotted site was analyzed. The results suggested that the ratios of carbon, oxygen, and nitrogen were similar to those of *N*-acetylglucosamine (Figure 7B). To gain further insights into the biochemical structure of the string-like substances surrounding the mutant, we prepared a culture supernatant of FK300Δ5bpΔ*icaB,* which was then vigorously shaken to determine whether any autoaggregation occurred without bacterial cells and also whether any tiny but distinct whitish autoaggregates would appear after shaking (Supplementary Figure 3). Such substances appeared in FK300Δ5bpΔ*icaB* culture supernatants but not in FK300 and FK300Δ5bp culture supernatants. We studied *in vitro* autoaggregation in the culture supernatant of FKΔ5bpΔ*icaB*, to analyze the inhibitory effect of several additives, including NaCl, ethylenediaminetetraacetic acid (EDTA), dispersin B, proteinase K, and DNase. Only dispersin B, a β-hexosaminidase that specifically hydrolyzes β-1,6-glycosidic bonds in acetylglucosamine polymers (Kaplan et al., 2003; Ramasubbu et al., 2005; Itoh et al., 2005), inhibited the appearance of aggregates *in vitro*. This finding strongly suggested that acetylglucosamine polymers were the major factor(s) involved in the autoaggregation of FK300Δ5bpΔ*icaB*.

**Figure 8:**
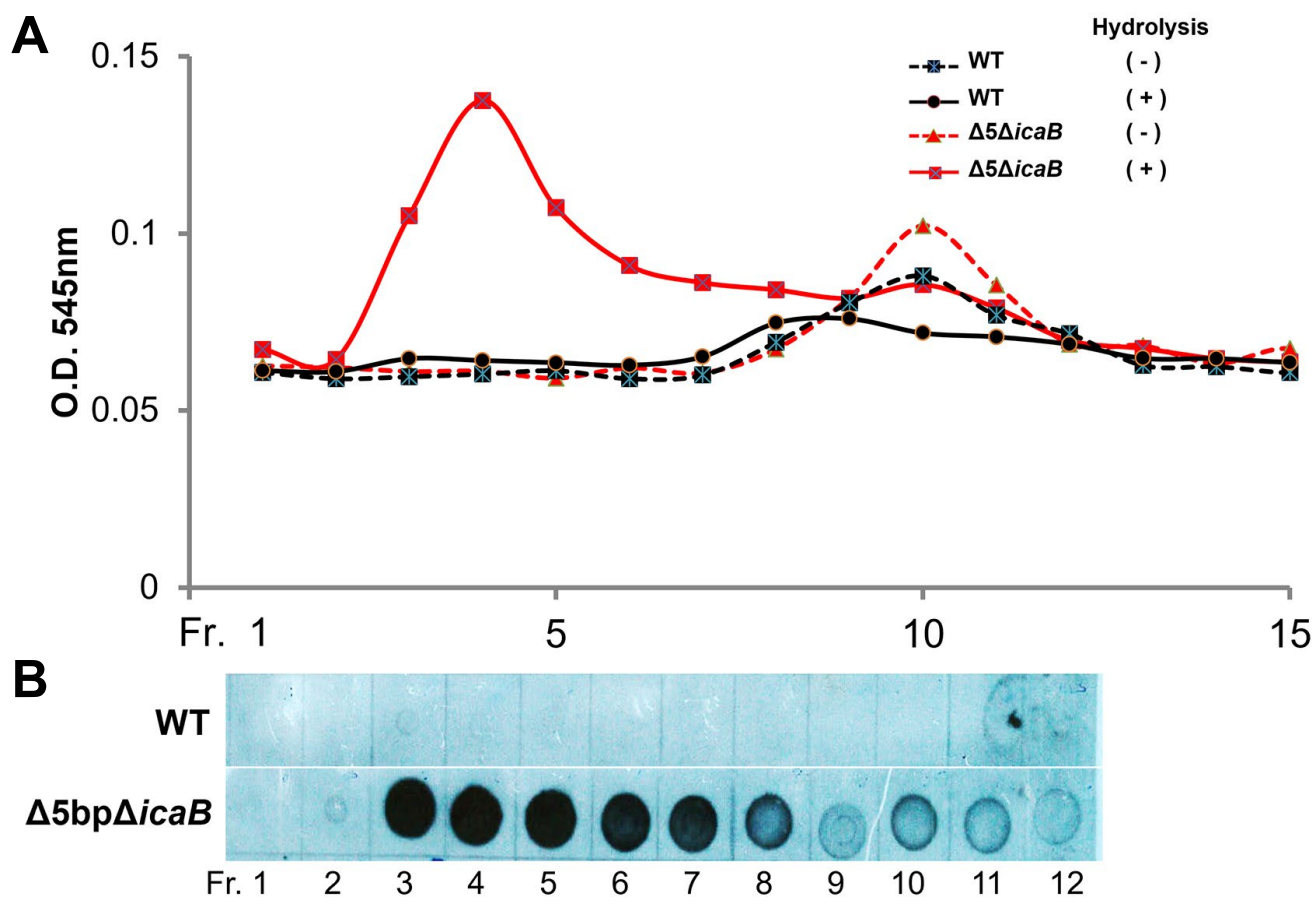
Gel permeation high performance liquid chromatography analyses of culture supernatants of FK300Δ5bpΔ*icaB* and WT. Gel permeation high performance liquid chromatography analyses of culture supernatants of FK300Δ5bpΔ*icaB* and WT. Concentrated culture supernatants of FK300Δ*5bp*Δ*icaB* and FK300 were subjected to gel permeation chromatography (GPC) using Shim-pack Diol-300 (500 × 7.9 mm) under water as the mobile phase with a flow rate 0.5 ml/min. Effluents were fractionated every 2 ml from fraction (Fr.) 1 to 21, 11 min after sample injection. Each fraction, with or without hydrolysis, was analyzed for amino sugar **(A)** using the Morgan-Elson colorimetric method and the PNAG **(B)** signal using dot blot with the rabbit anti-PNAG as described in the Methods section. WT; wild type strain FK300.

To further analyze the biochemistry of these polymers, the FK300Δ5bpΔ*icaB* culture supernatant was fractionated using gel filtration chromatography, and each fraction with or without hydrolysis was analyzed for amino sugar concentration. A broad peak appeared in the void-volume-column (fractions 3–8) of the HCl-hydrolyzed fractions, indicating that the polymers in the supernatant of FK300Δ5bpΔ*icaB* contained amino sugars (Figure 8A). Furthermore, dot blot analysis with antisera against PNAG clearly demonstrated that the corresponding fractions were positive for PNAG (Figure 8B). Collectively, these results strongly suggested that the factor(s) involved in the autoaggregation of FK300Δ5bpΔ*icaB* in the supernatant was an N-acetylglucosamine polymer.

**Figure 9:**
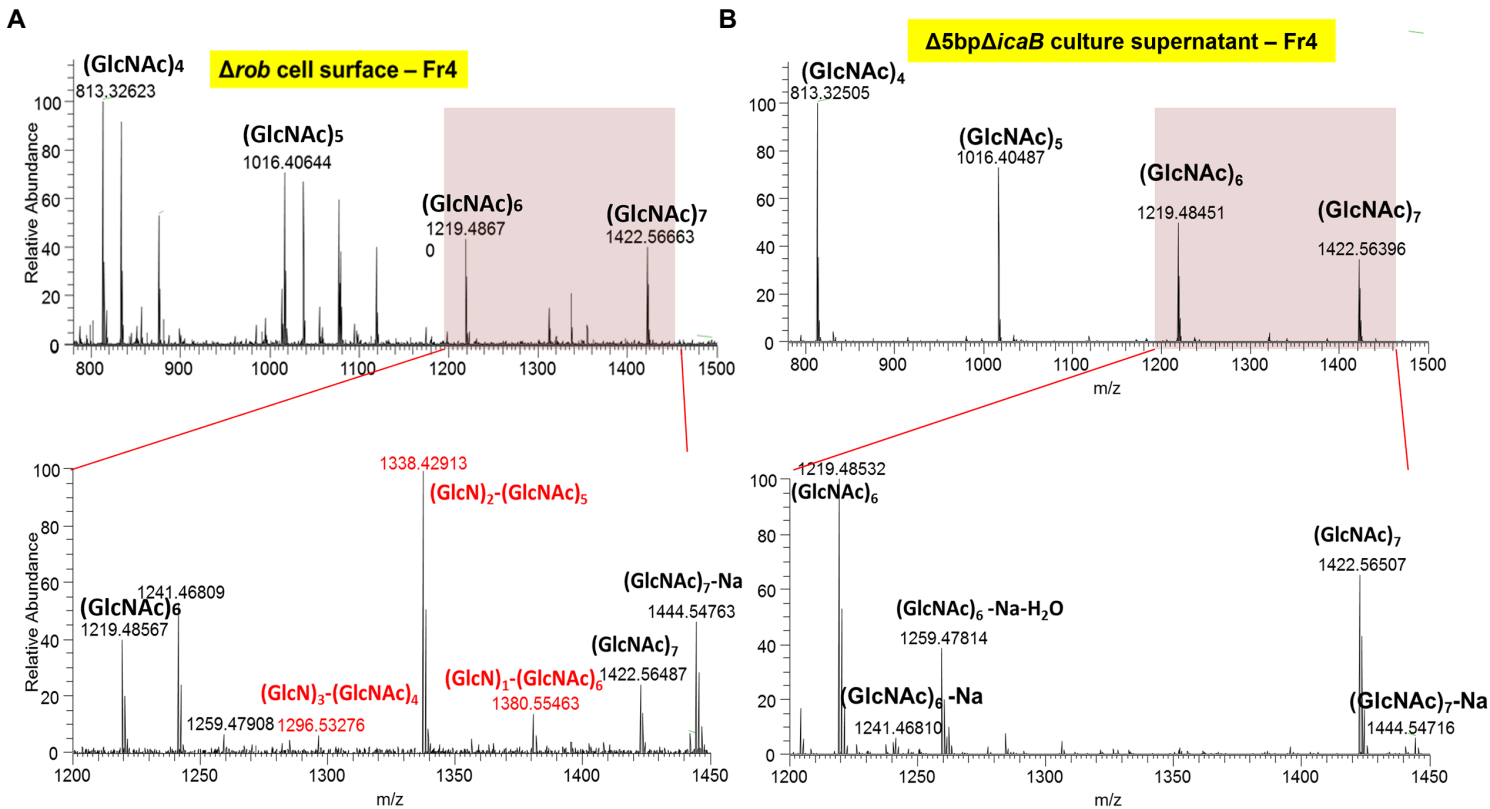
The typical ESI positive ion mass spectrum of PIA fractionated by GPC, showing the major mass differences to be deacetylation of poly-*N*-acetyl-D-glucosamine polymer. Typical ESI-positive ion mass spectrum of PNAG from FK300Δ5bpΔ*icaB* fractionated using GPC. Samples of cell surface FK300Δ*rob* **(A)** and culture supernatant FK300Δ5bpΔ*icaB* **(B)** from the collected fraction by GPC were analyzed using ESI-MS. MS analyses of authentic PNAG **(A)** showed fragmentation of polysaccharide homopolymers resulting in a series of ESI-MS of m/z = 203 Da, which corresponded to GlcNAc. Besides these spectra, several peaks defecting of m/z = 42 Da, which corresponded to deacetylation of [GlcNAc] polymer were observed in FK300Δ*rob* **(A)**. By contrast, the culture supernatant of autoaggregation inducing strain FK300Δ*5bp*Δ*icaB* **(B)** contained non deacetylated [GlcNAc] polymer. *N*-acetyl-D-glucosamine is expressed as GlcNAC, Glucosamine as GlcN. WT; wild type strain FK300.

*N*-acetylglucosamine polymers secreted from the cytosol of *S. aureus* undergo partial deacetylation by IcaB, which functions as a deacetylase (Vuong et al., 2004). The resulting extracellular positively charged deacetylated polymers interact with the negatively charged cell surface to form a biofilm matrix around the cells. FK300Δ5bpΔ*icaB* lacks IcaB deacetylase and is therefore unable to catalyze the deacetylation of *N*-acetylglucosamine polymers, which releases the polymer into the culture supernatant. Therefore, we investigated whether the polymers involved in the autoaggregation of FK300Δ5bpΔ*icaB* were deacetylated, by performing mass spectrometry (MS) analysis of the polymers. PNAG on the surface layer of the biofilm-producing strain, FK300Δ*rob*, was extracted and used as a control. MS analysis of PNAG gathered from the surface of FK300Δ*rob* cells showed MS signals with a regular interval of m/z = 203, which corresponded to the *N*-acetylglucosamine (GlcNAc) molecule. In addition, several signals corresponding to partially deacetylated polymers, such as (GlcN)_1_-(GlcNAc)_6_, (GlcN)_2_-(GlcNAc)_5_, and (GlcN)_3_-(GlcNAc)_4_ between (GlcNAc)_6_ and (GlcNAc)_7_, were observed (Figure 9A). By contrast, polymers recovered from the culture supernatant of FK300Δ5bpΔ*icaB* showed signals corresponding to (GlcNAc)_n_, but not those corresponding to deacetylated fragments (Figure 9B). These results clearly showed that the polymers in the supernatant of FK300Δ5bpΔ*icaB* were polymers of *N*-acetylglucosamine without deacetylation.

## Discussion

IcaB plays a crucial role in the biogenesis of extracellular *N*-acetylglucosamine polymers for adherent biofilm formation, by deacetylating *N*-acetylglucosamine polymers, which makes them electrostatically positive. These polymers then interact with the negatively charged bacterial cell surface *via* electrostatic interactions leading to PIA in *S. epidermidis* (Mack et al., 1996). Deletion of 5-bp in the icaR-icaA intergenic region leads to strong biofilm formation in *S. aureus* (Yu et al., 2017). Defective IcaB functioning leads to the production of fully acetylated *N*-acetylglucosamine polymers, which no longer interact with the cell surface and remain in the culture supernatant. Therefore, we assumed that FK300Δ5bpΔ*icaB* produces many fully acetylated poly-*N*-acetylglucosamine molecules and secretes them into the culture supernatant. Our study clearly demonstrated that the secretion of a large amount of poly-*N*-acetylglucosamine molecules without deacetylation played a direct role in the autoaggregation of *S. aureus* after vortexing.

Similar to the vortex-induced autoaggregation of *S. aureus* in this study, there was a report of vortex-induced aggregation in single-walled carbon nanotubes (Fernandes et al., 2017). Although the detailed physical mechanism is not known, the aggregates grew larger with increasing addition up to a certain concentration for single-walled carbon nanotubes, and then leveled off. This is similar to the present experimental results. Previous studies of carbon nanotubes suggested that aggregation was caused by the interaction of single-walled carbon nanotubes with each other due to the weakening of the shielding effect that prevented the tubes from aggregating. From this, it was inferred that the aggregation in this study was caused by the loss of positive charge due to deacetylation of PNAG caused by the dysfunction of *icaB,* which facilitated interaction of PNAG with each other.

In this study, Allelic exchange to create a 5-bp deficient strain confirmed the presence of an unselected secondary mutation in *icaB*. Three of the mutants with altered colony morphology each had a different mutation in *icaB*, and these were not clonal. This suggested that the 5-bp deletion mutation alone had a fitness cost under the conditions employed in mutagenesis. In Figure 3, clearly aberrant autoaggregation was observed in FK300Δ5bpΔ*icaB,* but not in FK300Δ*rob*Δ*icaB* and FK300Δ*icaR*Δ*icaB*. We analyzed the mRNA expression of *icaA* in the three double mutants and found that *ica* operon expression was considerably high in the FK300Δ5bp*ΔicaB* mutants (Figure 4). In agitation agglutination experiments with FK300Δ*icaR*Δ*icaB* and FK300ΔrobΔ*icaB* strains, the supernatant did not become clear, but small clumps were visible, suggesting that high expression of the *ica* operon is required to induce agglutination like FK300Δ5boΔ*icaB* in this study. Deletion of the 5-bp motif induced maximum expression of the *ica* operon. A previous study demonstrated that *rob* binds to the 5-bp motif (Yu et al., 2017). However, the magnitude of i*caA* expression in KF300Δ5bpΔ*icaB* was far greater than that in FK300ΔrobΔ*icaB*. This suggested that factor(s) other than *rob* were involved in 5-bp motif dependent *icaA* activation. The identity of these factor(s) remains to be explored.

Considering massive production of PNAG without deacetylation in culture supernatant of FK300Δ5bpΔ*icaB*, the signal in the culture supernatant in dot blot assay was weak (Figure 5A). This may be attributed to structural differences between fully acetylated poly-*N*-acetylglucosamine and PNAG used to generate antiserum. It remains unclear as to what extent the fully acetylated poly-*N*-acetylglucosamine reacted with the anti-PNAG serum used in this study. Therefore, it was not possible to ascertain quantitative differences between strains with and without *icaB* deletion *via* this comparison.

Biofilms are defined as adherent communities of microbial origin, represented by cells that adhere to substrates, interfaces, or each other, are embedded in a matrix of extracellular polymeric material, and exhibit an altered phenotype concerning growth, gene expression, and protein production. Cell aggregates formed in the absence of a surface (Trunk et al., 2018) and floating pellicles that form biofilms at the air-liquid interface (Vaccari et al., 2017) are also considered to be a type of biofilm. In this study, we found that strains with mutations in *icaB* caused the self-aggregation and settling as clumps by vortexing. This suggests that deacetylation of PNAG, a major component of staphylococcal biofilms, by *icaB* is necessary for biofilm adherence, but we have demonstrated that accumulation non-deacetylated PNAG formed huge of microbial aggregates as non-adherent biofilm. Non-stick biofilms have also been reported in previous papers with *Escherichia coli* (Schembri et al., 2003) and *Clostridium perfringens* (Obana et al., 2020).

Staphylococci are known to be the causative agent of endocarditis, and a high incidence of thromboembolism in mechanical heart valves, where non-physiological flow patterns cause platelet aggregation and free thrombus. It has been previously reported that mechanical valves cause eddy-like turbulence that carries thrombus downstream (Bluestein et al., 2000), and autoaggregation-causing strains such as *S. aureus* in this study may increase the risk of thrombus formation in patients implanted with mechanical valves. Autoaggregating mechanism of *S. aureus* demonstrated in this study may have implication to explain the phenomena of autoaggregation of *S.aureus* observed in chronic wounds (Kirketerp-Møller et al., 2008) and/or synovial fluid (Staats et al., 2021). In such a way, non-adherent biofilms of S. aureus could be potentially involved in pathogenicity and need further investigation.

In summary, we discovered abnormal autoaggregation of *S. aureus* mutants lacking both TATTT motif in the intergenic region between *icaR* and *ica* operon, and *icaB* function upon vortexing. This was illustrated by the production of a large amount of fully acetylated PNAG polymer in the culture supernatant, which induced autoaggregation by tiny physical stimuli and formed clumps. We propose this as an another type of biofilm, non-adherent biofilm.

### Genome sequencing

Genomic DNA extraction and whole-genome sequencing were performed as described previously27. The raw data reads of the 5-bp (*icaR*-*icaA* intergenic region) deletion mutants from S. aureus FK300, FK300Δ5bpBm 1, 2, and 3, have been deposited in the DDBJ/Sequence Read Archive under the accession numbers DRA013849.

## Data availability statement

The datasets presented in this study can be found in online repositories. The names of the repository/repositories and accession number(s) can be found at: https://www.ddbj.nig.ac.jp/, DRA013849.

## Author contributions

LY, JH, and MS conceived and designed the experimental studies. SK performed most experiments described in the paper and wrote the paper. JH analyzed the sequence data and supported experimental design and technique. LY provided assistance with the RNA isolation and qRT-PCR. SY provided assistance with the electron microscopy. IH provided assistance with Element analysis, Gel permeation HPLC and ESI-MS analysis. MS helped with writing and editing. All authors contributed to the article and approved the submitted version.

## Funding

The work was supported by Research Program on Emerging and Re-emerging infectious Diseases from the Japan Agency for Medical Research and Development (AMED) under grant number JP21fk0108604j0001, JP20gm1010001j0305, the Health and Labor Sciences Research Grant (21HA2009, 21KA1004) and JSPS KAKENHI Grant Number JP20K10268.

## Conflict of interest

The authors declare that the research was conducted in the absence of any commercial or financial relationships that could be construed as a potential conflict of interest.

## Publisher’s note

All claims expressed in this article are solely those of the authors and do not necessarily represent those of their affiliated organizations, or those of the publisher, the editors and the reviewers. Any product that may be evaluated in this article, or claim that may be made by its manufacturer, is not guaranteed or endorsed by the publisher.
