# Supplementary material for: Non-deacetylated poly-N-acetylglucosamine-hyperproducing Staphylococcus aureus undergoes immediate autoaggregation upon vortexing
Source: Front Microbiol. 2023 Jan 9;13:1101545. doi: 10.3389/fmicb.2022.1101545 (PMC9868172; doi:10.3389/fmicb.2022.1101545)
Supplement: Supplementary file 1 [file Presentation_1.PPTX]

## Slide 1
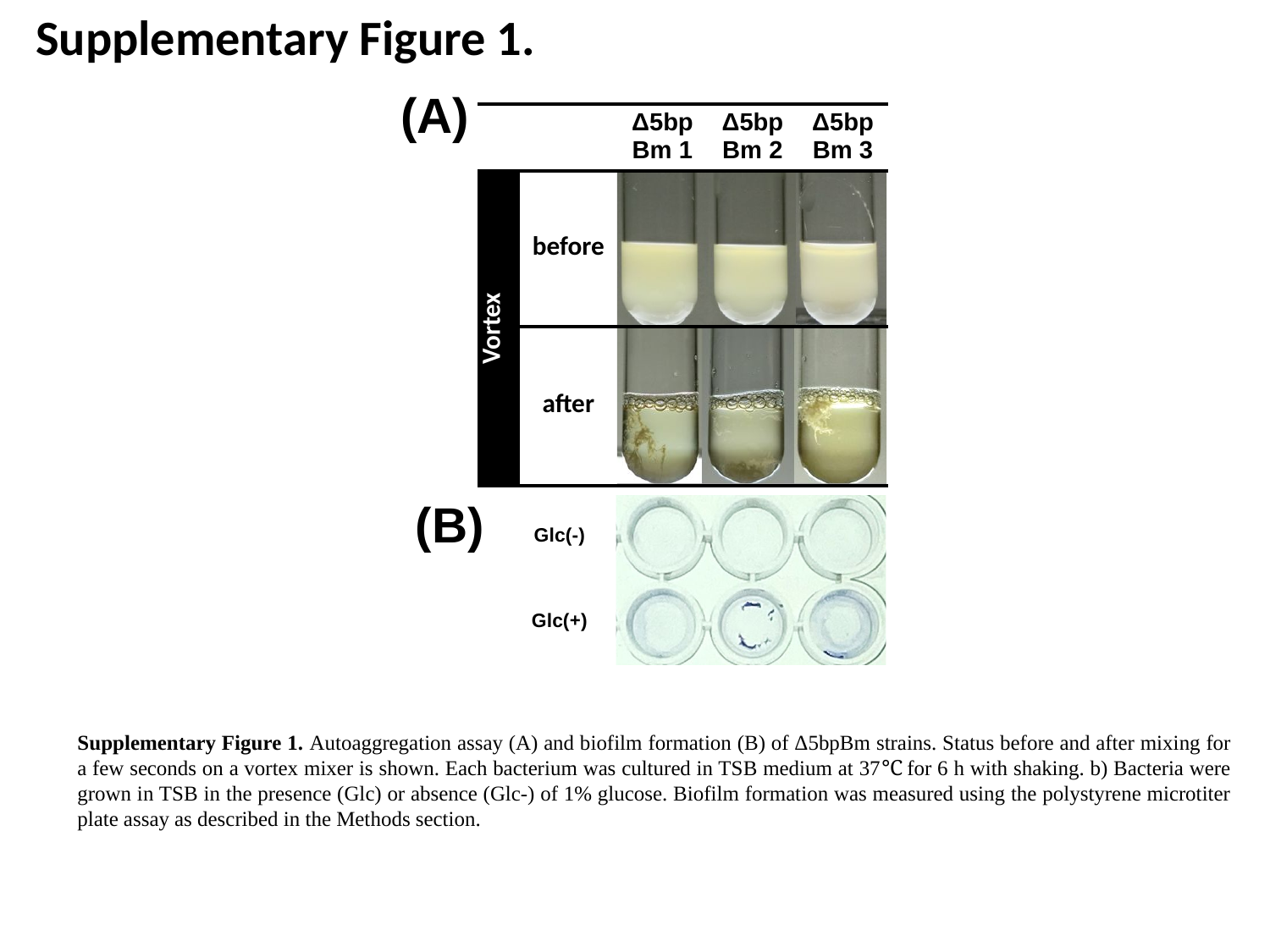

Supplementary Figure 1.
(A)
| | | Δ5bp Bm 1 | Δ5bp Bm 2 | Δ5bp Bm 3 |
| --- | --- | --- | --- | --- |
| Vortex | before | | | |
| | after | | | |
(B)
| Glc(-) | | | |
| --- | --- | --- | --- |
| Glc(+) | | | |
Supplementary Figure 1. Autoaggregation assay (A) and biofilm formation (B) of Δ5bpBm strains. Status before and after mixing for a few seconds on a vortex mixer is shown. Each bacterium was cultured in TSB medium at 37℃ for 6 h with shaking. b) Bacteria were grown in TSB in the presence (Glc) or absence (Glc-) of 1% glucose. Biofilm formation was measured using the polystyrene microtiter plate assay as described in the Methods section.

## Slide 2
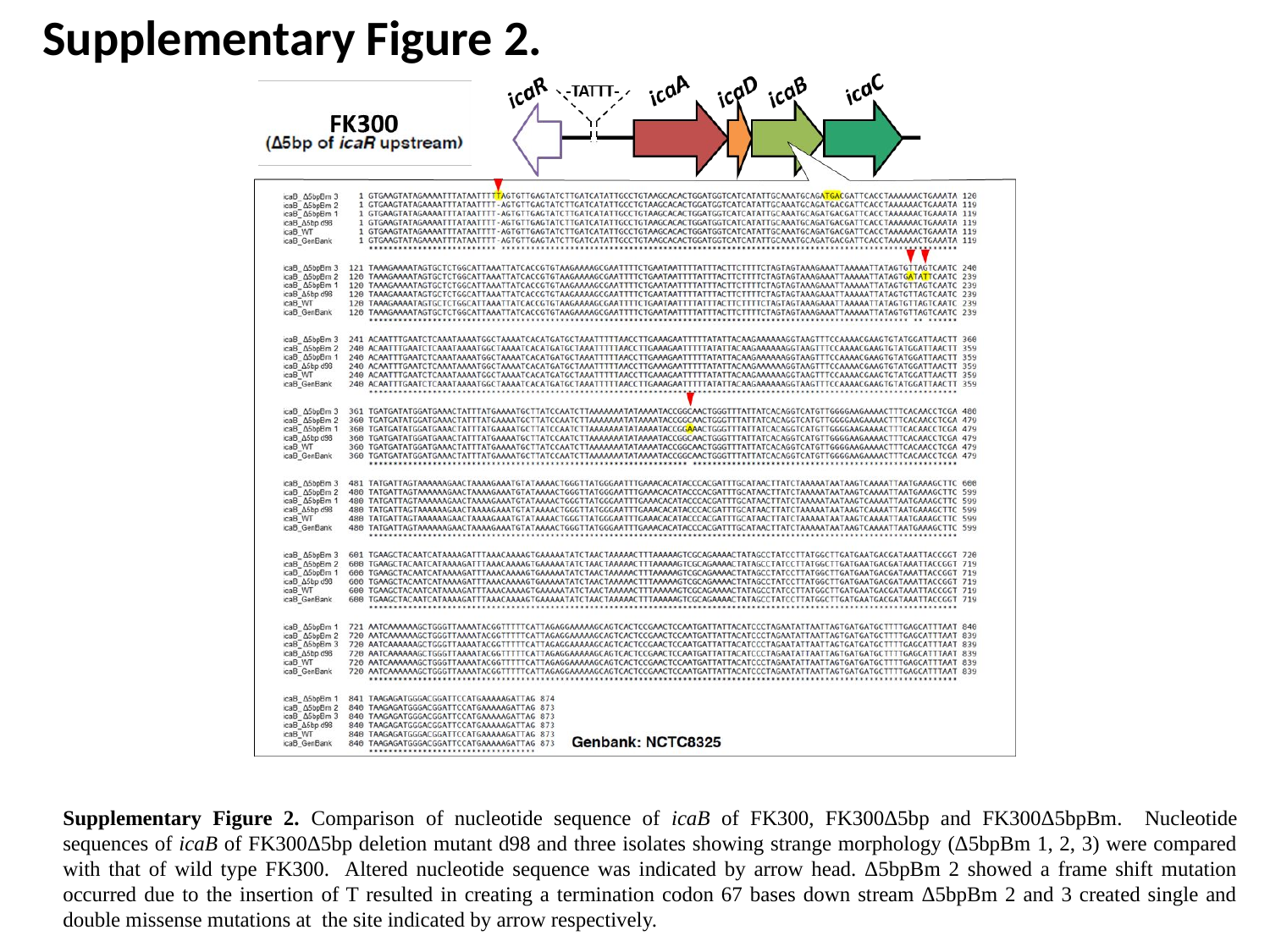

Supplementary Figure 2.
Supplementary Figure 2. Comparison of nucleotide sequence of icaB of FK300, FK300Δ5bp and FK300Δ5bpBm. Nucleotide sequences of icaB of FK300Δ5bp deletion mutant d98 and three isolates showing strange morphology (Δ5bpBm 1, 2, 3) were compared with that of wild type FK300. Altered nucleotide sequence was indicated by arrow head. Δ5bpBm 2 showed a frame shift mutation occurred due to the insertion of T resulted in creating a termination codon 67 bases down stream Δ5bpBm 2 and 3 created single and double missense mutations at the site indicated by arrow respectively.

## Slide 3
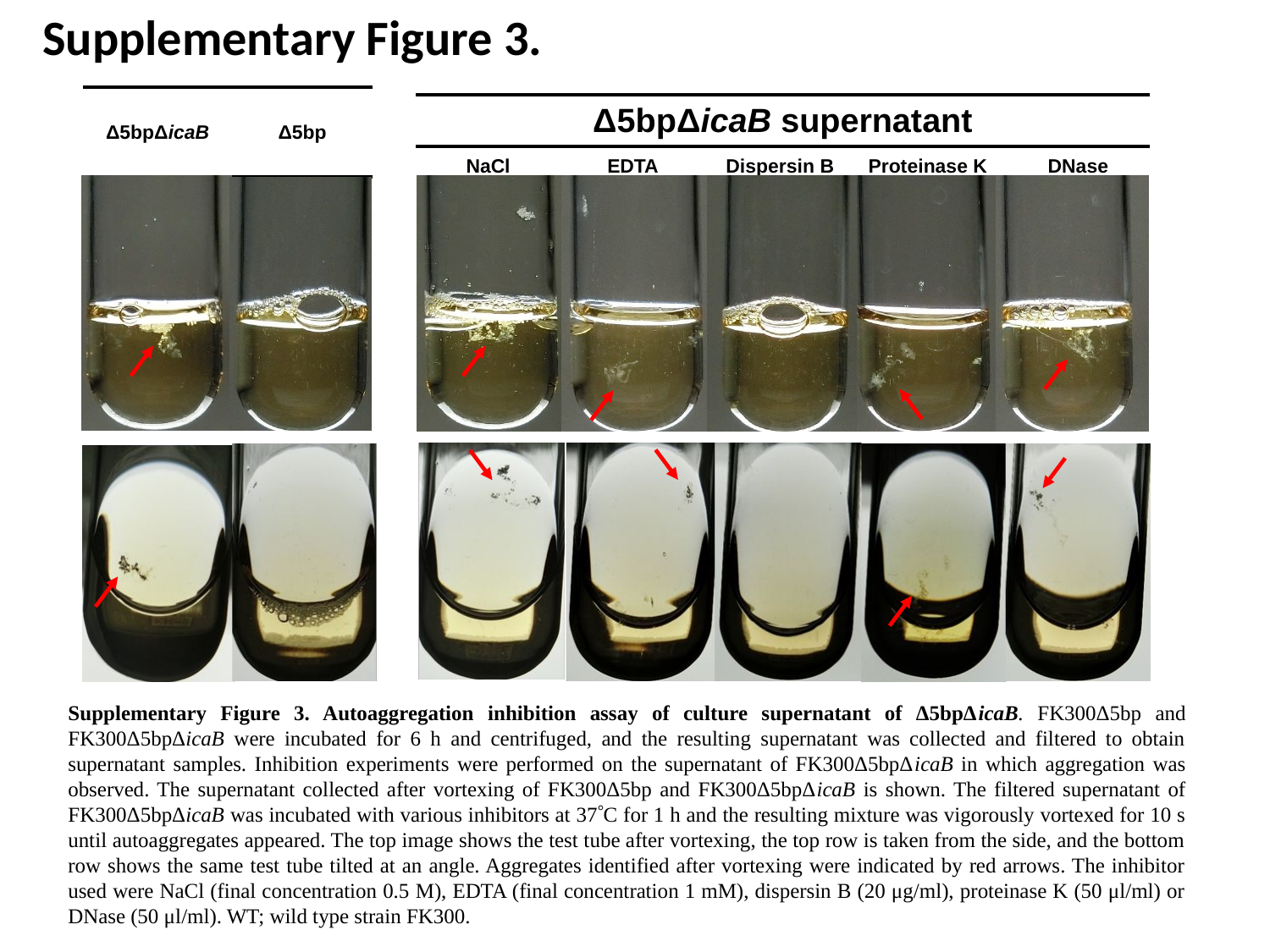

Supplementary Figure 3.
| Δ5bpΔicaB | Δ5bp |
| --- | --- |
| Δ5bpΔicaB supernatant | | | | |
| --- | --- | --- | --- | --- |
| NaCl | EDTA | Dispersin B | Proteinase K | DNase |
Supplementary Figure 3. Autoaggregation inhibition assay of culture supernatant of Δ5bpΔicaB. FK300Δ5bp and FK300Δ5bpΔicaB were incubated for 6 h and centrifuged, and the resulting supernatant was collected and filtered to obtain supernatant samples. Inhibition experiments were performed on the supernatant of FK300Δ5bpΔicaB in which aggregation was observed. The supernatant collected after vortexing of FK300Δ5bp and FK300Δ5bpΔicaB is shown. The filtered supernatant of FK300Δ5bpΔicaB was incubated with various inhibitors at 37°C for 1 h and the resulting mixture was vigorously vortexed for 10 s until autoaggregates appeared. The top image shows the test tube after vortexing, the top row is taken from the side, and the bottom row shows the same test tube tilted at an angle. Aggregates identified after vortexing were indicated by red arrows. The inhibitor used were NaCl (final concentration 0.5 M), EDTA (final concentration 1 mM), dispersin B (20 μg/ml), proteinase K (50 μl/ml) or DNase (50 μl/ml). WT; wild type strain FK300.
