# Supplementary material for: Non-deacetylated poly-N-acetylglucosamine-hyperproducing Staphylococcus aureus undergoes immediate autoaggregation upon vortexing
Source: Front Microbiol. 2023 Jan 9;13:1101545. doi: 10.3389/fmicb.2022.1101545 (PMC9868172; doi:10.3389/fmicb.2022.1101545)
Supplement: Supplementary file 2 [file Table_1.DOCX]

| **Supplementary Table 1.** Strains and plasmids used in the present study | |  |
| --- | --- | --- |
| Strain or plasmid | Relevant properties | Source or reference |
| Strains |  |  |
| *S. aureus* |  |  |
| FK300 | RsbU-repaired NCTC8235-4 as a standard strain | 1,2 |
| Δ5bpBm | FK300 Δ5bp *icaB* mutation | this study |
| RN4220 | Restriction-negative strain, NCTC8325-4 derivative | 3 |
| Δ5bp | FK300 Δ5bp | 4 |
| Δ*icaR* | FK300 Δ*icaR* | 4 |
| Δ*rob* | FK300 Δ*rob* | 4 |
| SK101 | RN4220 pKS102 | this study |
| SK102 | FK300Δ5bp pKS102 | this study |
| SK103 | FK300Δ*icaR* pKS102 | this study |
| SK104 | FK300Δ*rob* pKS102 | this study |
| SK105 | FK300Δ5bpΔ*icaB* | this study |
| SK106 | FK300Δ*icaR*Δ*icaB* | this study |
| SK107 | FK300Δ*rob*Δ*icaB* | this study |
| SK108 | RN4220　pKS103 | 5 |
| SK109 | FK300Δ5bpBm pKS103 | this study |
| SK110 | RN4220 pKS104 | this study |
| SK111 | FK300Δ5bpBm pKS104 | this study |
| SK112 | FK300Δ5bpΔ*icaB* pKS104 | this study |
| *E. coli* |  |  |
| DH5α | F-, Δ (*lacZYA* - *argF*) U169 (φ80d*lacZ* ΔM15), *recA1*, | TaKaRa |
| BL21 | Host for recombinant protein production | Novagen |
| SK113 | *endA*1, *hsdR*17, *supE*44, λ-, thi-1, *gyrA*96, *relA*1 | this study |
| SK113 | DH5α pKS101 | this study |
| SK114 | DH5α pKS102 | this study |
| SK115 | DH5α pKS104 | this study |
| Plasmids |  |  |
| pKFT | 5.8-kbp temperature-sensitive shuttle vector, Amp^r^ Tet^r^ in *E. coli*, Tet^r^ in *S. aureus* | 6 |
| pKAT | 4.0-kbp shuttle vector, Cat^r^ in *E. coli* and *S. aureus* | 7 |
| pGEM-T Easy | 3.0-kbp shuttle vector, Amp^r^ in *E. coli* | Promega |
| pKS101 | pGEM-T Easy containing regions upstream and downstream of the *icaB* gene | this study |
| pKS102 | pKFT containing regions upstream and downstream of the *icaB* gene | this study |
| pKS103 | pKAT containing the *icaR* coding region and promoter region | 4 |
| pKS104 | pKAT containing the *icaB* coding region, *sarA* promoter region, and *sodA* SD region | this study |

| **Supplementary Table 2.** Primers used in the present study | |
| --- | --- |
| Primer | Sequence (5′ to 3′) |
| Plasmid and strain construction |  |
| Δ*icaB*-F-F | ATTGAGTCGACAGTCGCACTC |
| Δ*icaB*-F-R | GGAATCCGTCCCATCTCTTATTCACGATTCTCTTCCTCTC |
| Δ*icaB*-R-F | GAGAGGAAGAGAATCGTGAATAAGAGATGGGACGGATTCC |
| Δ*icaB*-R-R | AGGTCTCGAGAAACGCTGTGTTG |
| *sarA* promoter F | CTCTAGAGATCCCCGGGCTGATATTTTTGACTAAACCAAATGC |
| *sarA* promoter R-*sodA* SD-*icaB*-F-R | TATAAATTTTCTATACTTCACAAATAATCATCCTCCTAAGATG |
| *sarA* promoter R-*sodA* SD-*icaB*-R-F | CATCTTAGGAGGATGATTATTTGTGAAGTATAGAAAATTTATA |
| icaB-SmaⅠ-R-R | CATCCCGGGCTAATCTTTTTCATGGAATCCGTCC |
|  |  |
| Quantitative PCR |  |
| *gyrB* for | AGGTCTTGGAGAAATGAATG |
| *gyrB* rev | CAAATGTTTGGTCCGCTT |
| *icaR* for | CGCCTGAGGAATTTTCTG |
| *icaR* rev | GGATGCTTTCAAATACCAAC |
| *icaA* for | AGTTGTCGACGTTGGCTAC |
| *icaA* rev | CCAAAGACCTCCCAATGT |
