# Supplementary material for: Non-deacetylated poly-N-acetylglucosamine-hyperproducing Staphylococcus aureus undergoes immediate autoaggregation upon vortexing
Source: Front Microbiol. 2023 Jan 9;13:1101545. doi: 10.3389/fmicb.2022.1101545 (PMC9868172; doi:10.3389/fmicb.2022.1101545)
Supplement: Supplementary file 3 [file Table_2.DOCX]

**References of supplemental material**

1. Giachino P, Engelmann S, Bischoff M. 2001. Sigma(B) activity depends on RsbU in *Staphylococcus aureus*. *J Bacteriol* 183(6):1843-52.

2. Herbert S, Ziebandt AK, Ohlsen K, Schäfer T, Hecker M, Albrecht D, Novick R, Götz F. 2010. Repair of global regulators in *Staphylococcus aureus* 8325 and comparative analysis with other clinical isolates. *Infect Immun* 78(6):2877-89.

3. Sambrook, J., Fritsch, E. F. & Maniatis, T. *Molecular Cloning: a Laboratory Manual* (Cold Spring Harbor Laboratory Press, Plainview, NY, 1989).

4. Yu L, Hisatsune J, Hayashi I, Tatsukawa N, Sato'o Y, Mizumachi E, Kato F, Hirakawa H, Pier GB, Sugai M. 2017. A Novel Repressor of the *ica* Locus Discovered in Clinically Isolated Super-Biofilm-Elaborating *Staphylococcus aureus*. *mBio* 8(1):e02282-16.

5. Cramton, S. E., Gerke, C., Schnell, N. F., Nichols, W. W. & Götz, F. 1999. The intercellular adhesion (*ica*) locus is present in *Staphylococcus aureus* and is required for biofilm formation. *Infect. Immun* 67**:**5427-33.

6. Kato, F. & Sugai, M. 2011. A simple method of markerless gene deletion in *Staphylococcus aureus*. *J. Microbiol. Methods* 87**:**76–81.

7. Kato, F. Induction of Staphylococcal bicomponent toxins; Exploration of inducers and their functions. (University of Tohoku. Sendai, Japan, 2004).
